# The complex geographies of telelactation and access to community breastfeeding support in the state of Ohio

**DOI:** 10.1371/journal.pone.0242457

**Published:** 2020-11-24

**Authors:** Tony H. Grubesic, Kelly M. Durbin

**Affiliations:** 1 Geoinformatics & Policy Analytics Laboratory, School of Information, University of Texas at Austin, Austin, TX, United States of America; 2 Childbirth International, Singapore, Singapore; Tel Aviv University, ISRAEL

## Abstract

The availability of breastfeeding support resources, including those provided by Baby-Friendly Hospitals, International Board Certified Lactation Consultants, breastfeeding counselors and educators, and volunteer-based mother-to-mother support organizations, such as La Leche League, are critically important for influencing breastfeeding initiation and continuation for the mother-child dyad. In addition, the emergence of community support options via information and communication technologies such as Skype and Facetime, social media (e.g., Facebook), and telelactation providers are providing mothers with a new range of support options that can help bridge geographic barriers to traditional community support. However, telelactation services that use information and communication technologies to connect breastfeeding mothers to remotely located breastfeeding experts require reliable, affordable, high-quality broadband connections to facilitate interaction between mothers and their support resources. The purpose of this paper is to explore the complex spatial landscape of virtual and face-to-face breastfeeding support options for mothers in the state of Ohio (U.S.), identifying barriers to support. Using a range of spatial and network analytics, the results suggest that a divide is emerging. While urban areas in Ohio benefit from both a density of face-to-face breastfeeding support resources and robust broadband options for engaging in telelactation, many rural areas of the state are lacking access to both. Policy implications and several potential strategies for mitigating these inequities are discussed.

## 1. Introduction

Breastfeeding is widely recognized as one of the most effective ways of ensuring child health and survival [1]. Breastfed children routinely have lower instances of obesity, diabetes, and improved cognitive function [2, 3]. Children are not the only ones to benefit, since mothers with a lifetime history of more than 12 months of lactation benefit from reduced instances of hypertension, diabetes, hyperlipidemia and cardiovascular disease [2]. While the relationship between breastfeeding and bonding remains an open research question [4], evidence suggests that breastfeeding can help solidify an emotional bond between mothers and infants [5, 6]. Beyond the benefits to the mother-child dyad, there are economic and environmental advantages [7, 8] to breastfeeding due to the cost of formula, inaccessibility of clean water [9–11], and the contribution to plastic waste by formula containers [12]. However, due to a host of personal, cultural, biomedical, socioeconomic and policy factors [13–20], breastfeeding is far from being a behavior which comes easy to mothers [21–24] or innate to the child [25]. For these reasons, it is critical that mothers receive the support they need to meet their feeding goals [26, 27], which may involve exclusive breastfeeding or mixed feeding–all in an effort to prompt the healthiest outcomes for the mother-child dyad.

The availability of community breastfeeding support plays an important role in breastfeeding continuation [28–30]. Grubesic and Durbin [31, 32] outline the four major forms of community resources that are generally available to mothers in the U.S.: 1) International board certified lactation counselors (IBCLCs) who provide clinical expertise on the mechanics of breastfeeding and any health-related challenges that may arise from breastfeeding for the mother and baby, 2) breastfeeding counselors and educators who provide non-clinical perspectives, education, guidance, and help with the mechanics (e.g., latch) of breastfeeding, 3) volunteer-based mother-to-mother support organizations such as Breastfeeding USA (BfUSA) or La Leche League (LLL) that provide mothers with information on breastfeeding best-practices and a forum for discussing the personal and sociocultural challenges of breastfeeding, 4) telelactation, social media (e.g., Facebook), and other platforms mediated by information and communication technologies (ICTs) such as Skype, or Facetime. This includes providers such as Pacify [33] or Maven [34], where breastfeeding support is available from IBCLCs via smartphone application or website. Lastly, it is also important to acknowledge that the special supplemental nutrition program for women, infants and children (WIC) also provides eligible families with education, resources, and assistance on breastfeeding [35, 36]. WIC operates in all 50 states, 34 Indian Tribal Organizations, and all of the U.S. territories.

The fundamental problem with these models of community breastfeeding support is that there may be a spatial mismatch between the location of mothers in need of support and the individuals or organizations that can provide it [31, 32, 37]. Specifically, while mothers located in larger urban areas may benefit from access to multiple IBCLCs, Baby-Friendly Hospitals, and an array of LLL or BfUSA meetings, mothers located in rural areas may not have any access to these support structures. Further, while telelactation options could provide help/support for mothers in more remote communities, these services require broadband connections that may (or may not) be available [38]. To be clear, the digital divide is alive and well in the United States. The FCC currently defines broadband as 25 Mbps downstream and 3 Mbps upstream and suggests that 18 million Americans are without broadband in 2020 [39]. However, recent work from Microsoft suggested that significantly more Americans (157.3 million) were not using the internet at broadband speeds [40].

The purpose of this paper is to explore the spatial landscape of community-based (i.e., face-to-face) and virtual breastfeeding support options in the state of Ohio. In particular, this work will identify communities where support options may be sparse and/or difficult to reach because of their location or the lack of adequate broadband infrastructure to facilitate telelactation. In addition, we explore the socioeconomic correlates that may be fueling this divide for breastfeeding support and discuss options for mitigating these inequities.

This work is important for three reasons. First, breastfeeding remains suboptimal in the United States and Ohio is a laggard. The Healthy People 2020 guidelines detailed in Tables 1 and 2, which are part of the U.S. government’s prevention agenda for building a healthier nation [41], suggest that the proportion of infants breastfed at 6 months and exclusively breastfed at 6 months in the United States are still below target levels. The story is worse for Ohio. While it meets the Healthy People 2020 targets for reduced formula supplementation and infants ever breastfed, Ohio ranks 36^th^ in exclusive breastfeeding at 6 months for all states and territories. Second, the deficit of high-quality breastfeeding support in rural and remote areas of the U.S. reveals the need for more telelactation options, but the persistence of the digital divide in rural America continues to stifle such efforts [42, 43]. Deepening our understanding of where these gaps may exist can help facilitate more proactive measures to provide the infrastructure required to support telelactation, and/or telemedicine more generally. Finally, the identification of locations for potential interventions, where more breastfeeding support options are required, can help stakeholders, policy-makers, and public health professionals to optimally allocate support resources under severe budget constraints in the current economic downturn [44]. More importantly, the framework detailed in this paper is generalizable to any location.

**Table 1:** Healthy people 2020 breastfeeding targets and realized rates for 2018.

Number	Objective	2006 Benchmark	Ohio	National	Healthy People 2020 Target
MICH-21	Increase the proportion of infants who are breastfed:				
MICH-21.1	Ever	74.0%	81.9%	83.2%	81.9%
MICH-21.2	At 6 months	43.5%	53.1%	57.6%	60.6%
MICH-21.3	After 1 year	22.7%	30.7%	35.9%	34.1%
MICH-21.4	Exclusively through 3 months	33.6%	44.4%	46.9%	46.2%
MICH-21.5	Exclusively through 6 months	14.1%	23.7%	24.9%	25.5%
MICH-22	Increase the proportion of employers that have worksite lactation programs	25.0%	-----	49.0%	38.0%
MICH-23	Reduce the proportion of breastfed newborns who receive formula supplementation within the first two days of life	24.2%	12.6%	17.2%	14.2%
MICH-24	Increase the proportion of live births that occur in in facilities that provide recommended care for lactating mothers and their babies	2.9%	-----	26.1%	8.1%

Target Met.

**Table 2:** 

RANK	State/Territory	Ever breastfed	Breastfeeding at 6 months	Breastfeeding at 12 months	Exclusive breastfeeding through 3 months	Exclusive breastfeeding through 6 months	Breastfed infants receiving formula before 2 days of age	Live births occuring at Baby-Friendly facilities, 2018
1	**Alaska**	93.1	69.2	49.7	65.3	42.1	11.4	3.4
2	**Vermont**	89.3	70.9	51.3	62.8	38	9.9	10.4
3	**Minnesota**	89.2	65.3	38.9	56.3	37.2	7.2	30.6
4	**Montana**	83.9	61.1	40.5	56.8	35.7	9.2	27.9
5	**Maine**	85.3	62.1	41.8	52.5	34.1	13.3	18.4
6	**Oregon**	89.4	72.5	51.7	57.8	33.4	13.4	52.6
7	**Hawaii**	90.6	65.6	47.2	54.9	32.9	17.3	12.1
8	**South Dakota**	83.3	62.6	42.7	54.3	32.2	11.7	4.9
9	**Indiana**	78.8	53.5	33	47.5	31.7	11.8	31
10	**Missouri**	82.3	57.8	33.1	52.7	31.3	14	13.2
11	**New Hampshire**	87.4	64.7	45.6	55.9	30.2	11.4	49.4
12	**Iowa**	81.5	51.4	30.2	51.6	29.5	8.4	8.1
13	**District of Columbia**	83	65.5	43.6	52.6	29.1	14	49
14	**North Dakota**	81.7	58.2	33.4	46.2	29.1	10.8	13.8
15	**Washington**	92.4	72.7	48.2	58.9	29.1	12.7	18.4
16	**Rhode Island**	81.4	49.6	30.9	47.9	28.9	18.3	86
17	**Wyoming**	90	59.4	38.6	56.8	28.8	9.4	2.4
18	**Idaho**	90.1	62.1	39	52.4	28.4	9.5	9.8
19	**Wisconsin**	82.2	59	39.3	48.8	28.3	15.6	16
20	**Utah**	89.7	62.5	40.8	49.7	27.8	20.1	8.6
21	**New Mexico**	87.7	59.8	35.1	53	27.6	11.5	54.3
22	**North Carolina**	84.9	58.8	33.2	48.1	27	15.6	37.6
23	**Massachusetts**	87.4	55.6	36.8	46.5	26.6	13.7	19
23	**Virginia**	81.7	62.5	39.3	45.6	26.6	20.9	12.7
25	**Puerto Rico**	85.9	47	29.8	48.4	26.5	19.6	1.1
26	**Arizona**	82.7	55.3	35.5	51.8	26.3	15.8	6.8
26	**California**	87.2	66.7	40.2	53	26.3	15.1	44.8
28	**Maryland**	91	66.8	41.1	50.1	26.2	19.1	18.2
29	**Kansas**	83.6	58.2	36.5	50.4	26.1	13.5	41.1
30	**Pennsylvania**	83.8	59.2	39	48.9	25.6	14.4	25
31	**Nebraska**	82.2	57	40.2	46.7	25.4	17.5	12.8
	**US National**^**§**^	83.2	57.6	35.9	46.9	24.9	17.2	26.1
32	**New Jersey**	82.8	57.6	36.1	40.6	24.4	25.7	18.9
32	**South Carolina**	76.4	45.1	28	42.7	24.4	15.2	41.7
34	**Texas**	85	56.6	35.2	48	24.1	18.3	20.1
35	**Michigan**	77.7	55.6	34.6	44.1	23.9	13.2	30.3
36	Ohio	81.9	53.1	30.7	44.4	23.7	12.6	16.5
37	**Connecticut**	86.3	59.6	39.1	45.5	23.6	20.3	46.3
37	**Delaware**	77.4	55.6	33.4	47.2	23.6	14.4	88.1
39	**Tennessee**	75.7	49.8	34.4	34.5	22.7	21.3	21.1
40	**Colorado**	90.9	63.9	40	57.2	22.4	10.6	48.9
41	**Georgia**	84	55.5	34.9	43.8	22.1	20.6	31.1
42	**Oklahoma**	75.9	49	31	44.2	21.6	16.8	21.7
43	**New York**	85.1	59.5	38.3	42.8	21.4	26.5	21.6
44	**Florida**	82.6	54	33.5	41.6	21.3	23.9	17.5
45	**Kentucky**	73.9	48.6	28.2	39.8	21.1	19.8	24.5
46	**Nevada**	83.5	49.9	30.6	44.1	20.8	23.7	16.3
47	**Alabama**	68.1	39.1	24.8	34.1	20.6	11.8	16.5
48	**Arkansas**	73.8	45.2	24.2	39	20.4	12.6	21.7
49	**Louisiana**	67	39	20.6	39.4	20.2	15.7	41.6
50	**West Virginia**	68.6	40.1	24.3	36.3	20.2	14.9	8.1
51	**US Virgin Islands**	83.9	51.9	33.1	31.6	19.9	27	0
52	**Illinois**	80.3	53	33.8	39.6	19.5	20.7	22.3
53	**Guam**	80.6	49	29.7	38.8	19.4	23.8	0
54	**Mississippi**	63.2	35.4	18.3	28.2	13	25.1	12.5

## 2. Background

### 2.1 Inequities in breastfeeding support

Emmott, Page and Myers [45] detail the three fundamental types of support, from a social epidemiological perspective: 1) *emotional*, 2) *informational*, and 3) *practical*. Emotional support takes many forms, but typically represents an empathetic connection between the supporter and the mother. Sometimes this connection is related to breastfeeding and its challenges, but other times it may represent a spectrum of general emotional support from family, friends or peers. Informational support involves the transfer of knowledge about breastfeeding from the supporter to the mother. This can take several forms, including breastfeeding promotion and encouragement, but recent work suggests that conflicting or inaccurate information can destabilize breastfeeding efforts [46]. Finally, practical support involves direct actions or interventions. For example, practical support includes friends or family providing childcare, helping with household chores, or any other activity that allows mothers to focus additional time and effort on breastfeeding [45]. A fourth type includes affirmational or conformational support [47], where peers provide assurances that breastfeeding efforts are going well. Finally, network support helps to build connections across a social network so that emotional, informational, practical, and affirmational support are more accessible [48]. This may include, but is not limited to, peer supporters introducing breastfeeding mothers to one another, or the use of Facebook groups to connect geographically distant mothers–both of which are forms of support that broadband and related ICTs help facilitate.

Depending on the ecological conditions influencing the mother-child dyad (e.g., socioeconomic, sociocultural, etc.) some mothers may have better *access* to support than others. In this context, ‘access’ is a term that is filled with complexities, some obvious–others nuanced. Specifically, access is often conflated with accessibility, availability, and/or equity. The differences in these terms are particularly important when considering the *geographic* dimensions of breastfeeding and breastfeeding support equity [15, 31, 37, 49]. Consider the following: 1) *access* typically refers to the journey between a household and a location where breastfeeding support can be obtained. As distance increases, access generally decreases because the effort required to travel between two distant locations grows [50]; 2) *accessibility* refers to the options available to a person seeking support once the initial trip is accomplished [51]. For example, if breastfeeding support options at a particular destination cost money (e.g., from a certified lactation consultant or IBCLC), accessibility may decrease for people without the means to pay for these services [52]. If the breastfeeding support options are free to all (e.g., mother-to-mother support such as LLL), accessibility improves; 3) *availability* generally refers to the count and range of options available to a person seeking support. High-availability communities often include a WIC breastfeeding counselor, LLL and/or Breastfeeding USA (BfUSA) meetings, as well as multiple IBCLCs. Low-availability communities might only include a WIC office; 4) spatial *equity* refers to the distribution of services in a region and whether or not all members of a society can obtain them regardless of race, economic status, place of residence or culture [31]. Lastly, there are temporal constraints that intermingle with some of these factors. For example, public transport options (e.g., bus, train) may not be operating when a mother needs to access local support resources. The overall accessibility of local resources is reduced when mother-to-mother support meetings are only held once or twice a month. In addition, the locations of these meetings may change (or close), forcing mothers seeking support to reconfigure their journey, if that journey is still possible.

### 2.2 Telelactation and the digital divide

By definition, telelactation refers to services that connect breastfeeding mothers to remotely located IBCLCs using information and communication technologies [53]. As detailed by Uscher-Pines et al., [54] there are a number of offerings that constitute telelactation: 1) large, direct to consumer telemedicine companies (e.g., Pacify), 2) startup companies exclusively focused on telehealth for women (e.g., Nest Collaborative), and 3) lactation consultants who offer virtual visits via video platforms such as Skype and Facetime.

There are several, notable advantages to telelactation that improve upon traditional, face-to-face support sessions, including convenience and cost. Where the former is concerned, mothers can obtain support from the comfort of their own homes, assuming that they have an internet connection and a device that supports virtual and/or video interactions. This is particularly valuable for mothers located in rural or remote locations, where lactation professionals may not be available; or for mothers who may have physical challenges (e.g., recovering from birth, a handicap, etc.) which makes travel difficult or impossible. A second advantage is that telelactation services tend to cost less than in-person visits [54], although this will certainly vary by location and provider (especially entrepreneurial IBCLCs). Lastly, Uscher-Pines et al., suggest that the very nature of telelactation, which is decidedly “hands-off,” helps mothers develop their skills independently and improving their self-efficacy.

Recent research confirms that telelactation is helping meet the needs of rural mothers. For example, Kapinos et al. [53] interviewed 47 rural mothers who participated in telelactation activities in Pennsylvania. 91% of the users (43/47) expressed satisfaction with the help they received. Additional work from Uscher-Pines [55] evaluates the feasibility and impact of telelactation on breastfeeding duration among rural women. Although the trial was not sufficiently powered to detect statistically significant differences in breastfeeding rates across study arms (telelactation [intervention] and usual care [control]), women engaged with telelactation were found to be breastfeeding at higher rates when compared to the control group.

While this early evidence on the effectiveness of telelactation is promising, several challenges remain. For example, work by Demirci et al. [56] suggests that some mothers are reluctant to conduct video calls with an unknown provider or may prefer using community-based breastfeeding resources. Mothers can also receive inconsistent advice, especially when obtaining information from multiple IBCLCs over the course of several sessions [54], but this is not unique to telelactation. In addition, there can be technical issues associated with limited internet connections that impact mothers seeking telelactation support, which can also include mothers who have limited (or no) training in using computing devices.

The use of such computing devices (e.g., laptop, tablet, mobile phone, etc.) is important because they enable telelactation, but ICTs and broadband are the platforms that facilitate it. As a result, access to broadband plays an important role for mothers seeking telelactation support. Unfortunately, access to broadband in the United States and elsewhere is radically uneven. This uneven access is routinely referred to as the “digital divide” [57]. The divide manifests in different ways for different groups. This includes divides across racial lines [58, 59], income [60, 61], social groups [62], age [63] and geography [64, 65], among others. The digital divide is particularly acute between urban/suburban and rural/remote areas [66–68]. Specifically, urban residents can often choose from several providers and platforms (e.g., cable, fiber), enjoy higher quality of service [69] and benefit from the latest technological upgrades [70]. If broadband *is* available in a rural or remote area, subscribers often suffer from monopolistic or duopolistic broadband markets [71]–experiencing higher costs, lower quality of service and slow (or non-existent) technology upgrade cycles [72–75].

Given the major health and social benefits of breastfeeding, it is critically important to protect, promote and support breastfeeding efforts in all communities [76], ensuring the availability of support regardless of race, economic status, place of residence or culture. Given the deficit of high-quality breastfeeding support in many rural and remote areas of the U.S., there is a growing need for telelactation services. However, the persistence of the digital divide in rural America continues to stifle such efforts. Identifying where these gaps may exist can help facilitate more proactive measures to provide the infrastructure required to support telelactation. In the next several sections, we detail a suite of methods and allied data for deepening our understanding of telelactation and access to community breastfeeding support in the state of Ohio.

## 3. Study area, data and methods

### 3.1 Study area

**Fig 1:**
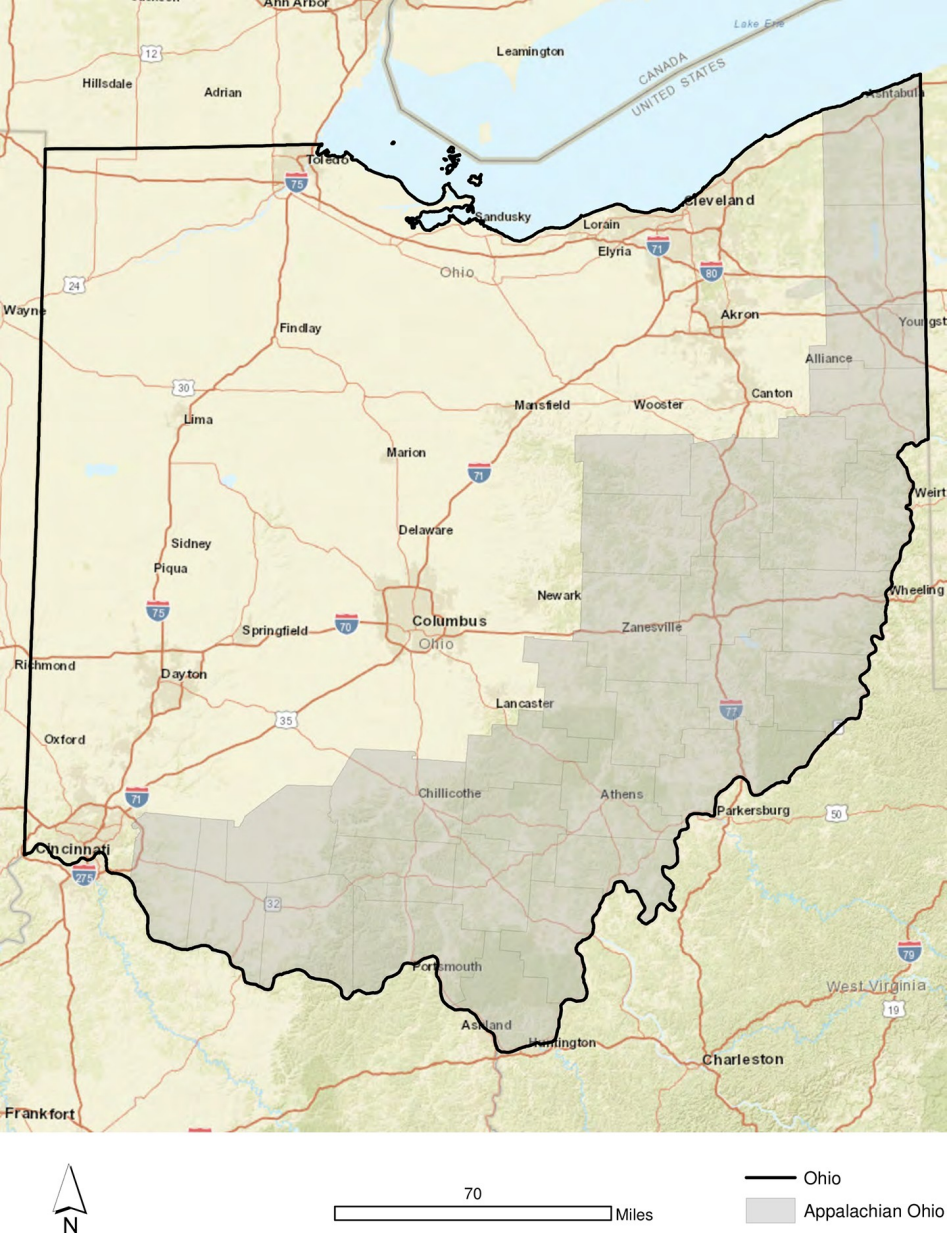
Appalachian Ohio.

Ohio is an interesting location for exploring access to breastfeeding support resources. It is the seventh most populous state in the U.S., with approximately 11.7 million residents (Census, 2019) (Fig 1). Ohio’s interstate corridors (I-70, I-71, I-75, I-77) are the most densely settled portion of the state—home to the larger cities of Cleveland, Columbus, Cincinnati, Dayton, Toledo and Akron. However, large swaths of northwestern and southeastern Ohio are rural. Much of Northwest Ohio focuses on industrial agriculture (e.g., corn and soybeans), while the bulk of southeastern Ohio corresponds to Appalachian Ohio, a region where the economy is based on resource extraction (e.g., coal) or materials processing. During 2020, the Appalachian Regional Commission [77] designated 4 of the counties in Ohio as economically “distressed” and 14 counties as “at risk” during 2020. These counties are frequent targets for federal, state and local partnerships seeking to improve the health and well-being of the local residents. The demographic profile for Ohio in 2019 is 81.9% white, 13% black, 3.9% Hispanic and 2.5% Asian. 13.9% of its population lives in poverty and 79.7% of households report a broadband internet subscription [78]. In many ways, Ohio is a microcosm of the United States–a representative mix of urban/rural, affluent/impoverished, and a somewhat diverse demographic profile.

### 3.2 Data

The data used for analysis in this paper come from a variety of sources, all of which are summarized and referenced in Table 3 [79–87]. Although most of these data are self-explanatory, there is nuance for several variables worth detailing. First, all of these data are dynamic, changing (sometimes rapidly), over time. For example, new IBCLCs may emerge (or disappear) in Ohio at any time. The same can be said for LLL and BfUSA meetings. Thus, at best, these data represent a snapshot of the community breastfeeding support landscape for 2019.

**Table 3:** Data sources.

Description	Details	Source	Reference
*Community Breastfeeding Resources*			
Baby Friendly Hospitals	*N* = 11	Baby-Friendly Hospital Initiative (2019)	[75]
IBCLCs	*N* = 196	International Lactation Consultant Association (2019)	[76]
La Leche League Meetings	*N* = 52	La Leche League International (2019)	[77]
Breastfeeding USA Meetings	*N* = 8	Breastfeeding USA (2019)	[78]
Women, Infant, and Children (WIC) Clinics	*N* = 199	United States Department of Agriculture/Ohio Department of Health (2019)	[79]
*Geographical and Ecological Data*			
Ohio County Basefile	*N* = 88	United States Census Bureau (2018)	[80]
Ohio Street Network	*N* = 478,064	Environmental Systems Research Institute (2019)	[81]
Tract-Level Demographic/Socioeconomic Data	*N* = 2,948*	Environmental Systems Research Institute (2019)	[82]
Ohio Broadband Data	*N* = 2,948*	Federal Communications Commission (2019)	[83]

* Some of these tracts were eventually removed from the analysis because they were part of wilderness areas large water areas or had no significant population associated with them.

Second, the diversity index used in this paper, which falls under the tract-level demographic/socioeconomic data, is a value that ranges between 0 and 100 that represents the likelihood that two persons, chosen at random from the same Census tract belong to different racial or ethnic groups. If the entire population of the tract belongs to the same race and ethnic group, the diversity index = 0. A tract trends toward 100 when the population is more evenly divided across race and ethnic groups. Esri [88] uses seven race groups: six single-race groups (White, Black, American Indian, Asian, Pacific Islander, Some Other Race) and one multiple-race group (two or more races). Each race group is divided into two ethnic groups, Hispanic and non-Hispanic.

Third, the data associated with broadband provision for Ohio [87] reflects provider information collected and processed in December 2017, but made available in 2019. This is typical for FCC broadband data, where data releases are lagged. While the data are certainly *not* perfect, the Form 477 data reported by providers to the FCC remains the only comprehensive, publicly available data source on broadband provision and speed in the United States [38, 64, 89]. More importantly, because of the duopolistic structure of broadband provision in the United States [71], change in the provision landscape is slow, especially in Appalachian Ohio [69], and these data remain representative. In this instance, the broadband data represent fixed internet access service connections per 1,000 households. We focus on households that have connections of at least 10 Mbps downstream and 1 Mbps upstream. While this does not currently qualify as “broadband” according to the FCC guidelines, which is currently 25 Mbps downstream and 3 Mbps upstream, the 10/1 Mbps connections are sufficient to provide the minimum bandwidth required to support streaming video (in both directions) for telelactation support. It is also important to note that the 10/1 Mbps connections represent the “floor” of these connection speeds. It is likely that many customers in this group enjoy true broadband speeds at 25/3 Mbps or higher. The 10/1 connections are divided into tiers for analysis, corresponding to connection densities. For example, 20% or less of the households in Tier 1 have 10/1 connections, while 60–80% of the households in Tier 4 have 10/1 connections.

### 3.3 Methods

This analysis focuses on household broadband connectivity tiers of 10/1 Mbps (Table 4) and the measurement variables corresponding to the demographic and socioeconomic data detailed in Table 5. Again, the overarching goal is to identify locations throughout Ohio where breastfeeding support resources may be sparse and/or difficult to reach because of their location or the lack of adequate broadband infrastructure to facilitate telelactation. Census tracts that fit this profile will be flagged either as Stage 1 or 2 intervention locations. Stage 1 locations will be de-prioritized for intervention because their combination of physical access and/or broadband service to facilitate telelactation options is lagging, but not critically weak. Stage 2 locations will have more severe constraints for accessing breastfeeding support, exhibiting poor physical proximity and/or a lower density of 10/1 broadband connections.

**Table 4:** Fixed residential broadband connections per 1,000 households by census tract: Ohio (2017).

Broadband Connection	Tier	Connections per 1000 Households	Tract Count
At least 10 Mbps downstream and 1 Mbps upstream			
	0	x = 0	33 (1.12%)
	1	0 < x < = 200	50 (1.70%)
	2	200 < x < = 400	314 (10.66%)
	3	400 < x < = 600	800 (27.13%)
	4	600 < x < = 800	1170 (39.69%)
	5	800 < x	581 (19.70%)

x = connections per 1000 households in a given tract.

**Table 5:** Descriptive statistics for demographic and socioeconomic indicators for Ohio tracts (N = 2,915).

Variable	Minimum	Maximum	*M* (*SD*)
Population	257	22520	4023.43 (1949.03)
Unemployment Rate	0	43.4	5.53 (4.89)
Diversity Index	1.5	88.5	29.42 (20.08)
Median Household Income	$9,680.00	$200,001.00	$55,121.51 ($25,109.50)
Housing Affordability Index	0	350	171.79 (47.87)
Wealth Index	9	456	77.48 (57.99)
% Bachelors or Graduate Degree	0	86.58	26.14 (17.99)
Relative Maximum Distance	0.055	2.849	0.636 (0.473)
Average Maximum Distance	0.013	0.0712	0.159 (0.118)

A one-way analysis of variance (ANOVA) provides an avenue for comparing the means of the measurement variables (Table 5) with respect to each of the broadband connectivity tiers (Table 4). Large differences between the means, especially along the traditional fissures associated with the digital divide (i.e., income, education, race), might suggest inequities in the distribution of broadband connections (and telelactation opportunities) throughout Ohio. Because the data in Ohio exhibit heteroscedasticity (i.e., the connectivity tiers have different standard deviations), we use Welch’s ANOVA [90] and the Games-Howell post-hoc test [91]. All statistical analysis was performed in Jamovi v. 1.2.19.0 [92].

Access to community breastfeeding support resources (Table 3) is calculated using network shortest paths. Specifically, the centroid of each Census tract (a geographically bounded administrative unit used for collecting population data, typically between 1,200 and 8,000 people) is connected to its nearest node on the high-resolution Ohio street network. Next, we use the Dijkstra’s Shortest Path algorithm [93] to identify the total distance required to travel from the tract centroid to the nearest LLL or BfUSA meeting, Baby-Friendly Hospital, IBCLC, and WIC clinic. The sum of these shortest paths (in kilometers) represents the aggregate access score for each tract (the smaller, the better). Each individual shortest path value can also be transformed into a relative access measure. This is calculated by dividing each tract’s shortest path value by the maximum shortest path value for all tracts. For example, if Tract 1 is 100 km distant from its nearest Baby-Friendly Hospital and Tract 2 is 120 km distant, one divides values for Tracts 1 and 2 by the maximum known value (Tract 23: 160 miles), yielding a relative access score of 0.625 for Tract 1, 0.750 for Tract 2, and 1.00 for Tract 3. Ultimately, this measure can then be used to evaluate the access score for each tract, relative to its peers. Again, lower values suggest higher levels of access. Derivative measures associated with the *sum of relative maximum distances* can also be used for evaluation.

Although tracts are relatively small geographic units, the use of tract centroids for summarizing the geographic distribution of access to breastfeeding support resources is somewhat limiting. The interstitial spaces between these representative geographic points may exhibit increased (or decreased) levels of access to breastfeeding support resources depending on the morphology of the tract itself. In short, some tracts are bigger than others and using a centroid may not capture the differences in access for an entire tract. We employ kriging to estimate these nuances in access [94]. In its most rudimentary form, kriging uses least-squares regression for estimating and explaining variations of a surface. One advantage to kriging is that it leverages information from surrounding locations to provide information about an unsampled point on a surface. It can also minimize estimation variance and provide a measure of uncertainty for each estimate [95]. There are many different approaches to kriging. For example, *simple kriging* assumes that the trend component is constant and that the mean is known. For *ordinary kriging*, it is assumed that the mean is an unknown, but that it is constant in the local neighborhood of each estimation point. This is helpful because there is no need to estimate a first-order trend–the mean is estimated from nearby data, only. Because of this, the surface estimates are not as sensitive to nonstationarity, which is ideal for this application. Thus, ordinary kriging is used to estimate the surface associated with the sum of the relative maximum distances to breastfeeding support for Ohio. Higher values will represent a longer relative distance required to access support resources. All geocomputation and visualization tasks were performed in ArcGIS v. 10.7.1.

## 4. Results

### 4.1 Geovisualization

**Fig 2:**
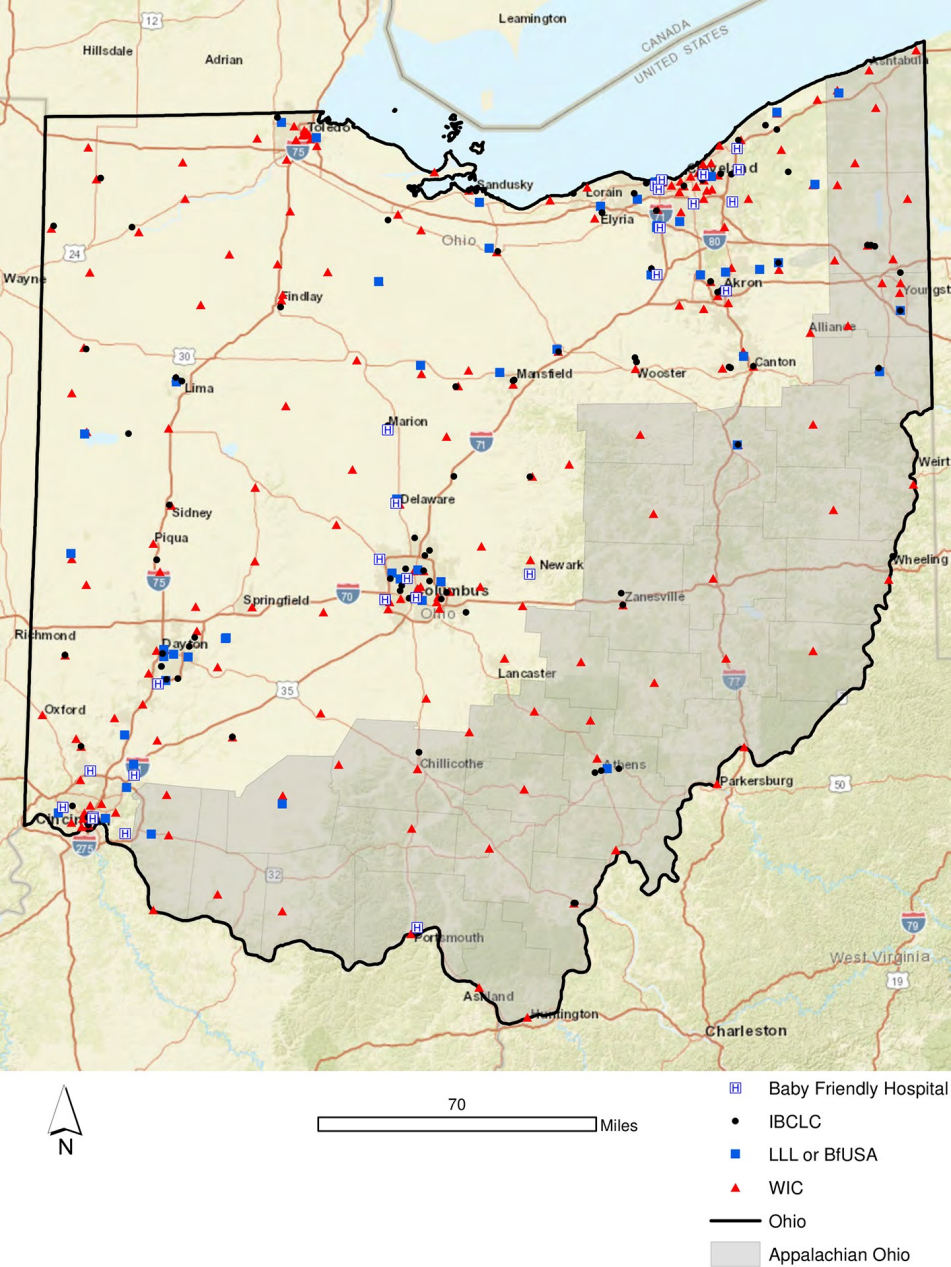
Breastfeeding support options available in the state of Ohio, 2019.

**Fig 3:**
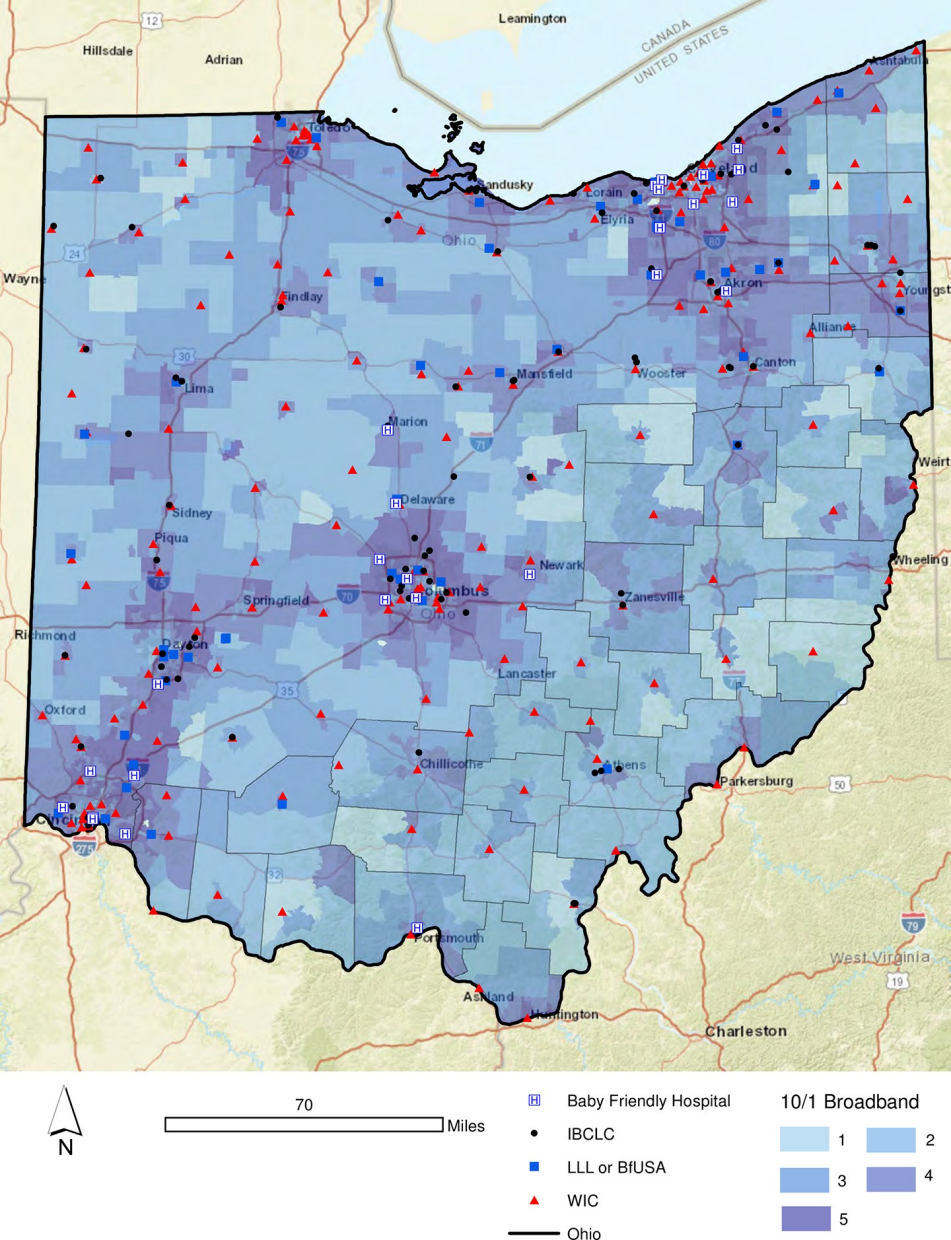
Broadband connection density by census tract, Ohio 2017.

Fig 2 illustrates the array of breastfeeding support options available in the state of Ohio, including Baby-Friendly Hospitals, LLL and Breastfeeding USA meetings, practicing IBCLCs and all of the WIC offices in the state. As one might expect, the larger urban areas exhibited a greater count of local support resources than the more rural areas of Appalachian Ohio and Northwest Ohio. That said, it is important to note that each county within the state has at least one WIC office. Fig 3 extends the geovisualization to include the 10/1 Mbps broadband tiers detailed in Table 4. For example, Tier 5 indicates that more than 80% of households (at least 800 per 1,000) households in the tract have broadband connections speeds that meet the 10/1 threshold. Again, while the highest density of 10/1 connections are located along the major interstate corridors in Ohio, some of the more geographically remote cities in Ohio also exhibited faster connection speeds, including Athens and Chillicothe (both of which are located in Appalachian Ohio). However, there is a clear gap in provision densities throughout Appalachian Ohio and portions of Northwestern Ohio (Fig 3).

### 4.2. One-way ANOVA

**Fig 4:**
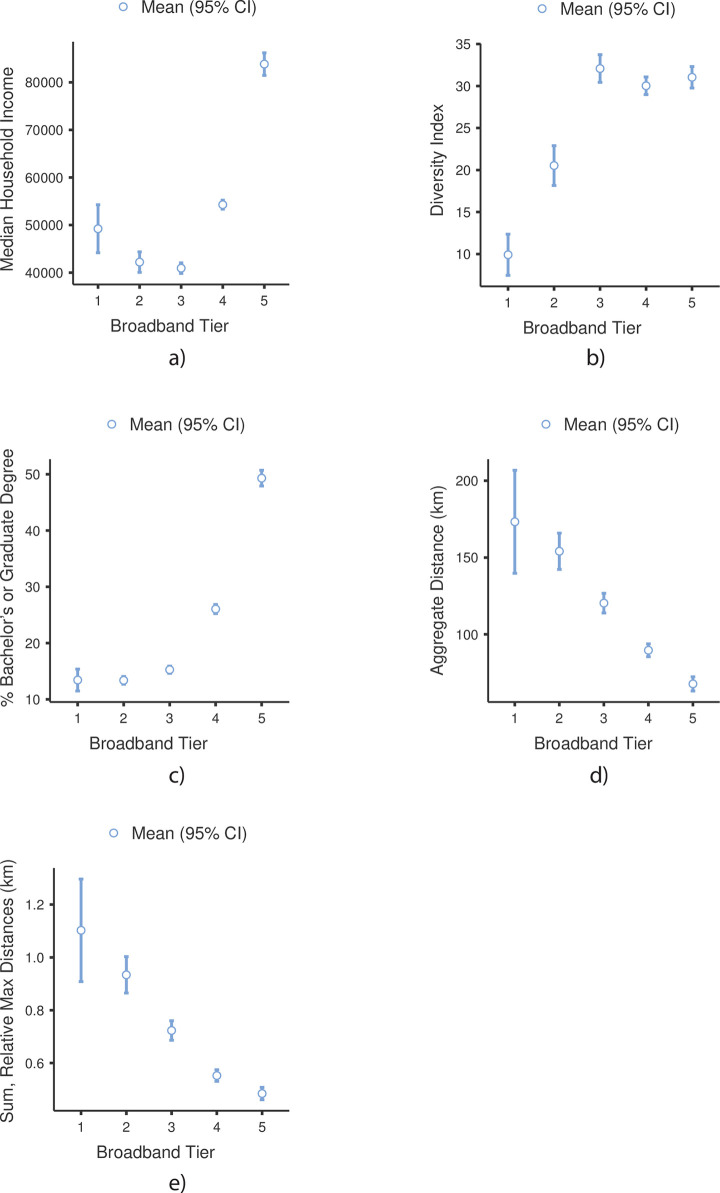
Welch’s ANOVA test results.

The one-way ANOVAs were used to explore the demographic and socioeconomic differences in Ohio tracts with at least 10/1 Mbps connection speeds and their associated broadband connection density tiers (1,2,3,4, and 5) (Table 4). The first test was used to determine if there were significant differences in median household income between the broadband tiers. The Welch’s ANOVA indicated significant group differences, *Welch’s F*(4, 325) = 291, *p* < 0.001. Pairwise comparisons of the means using the Games-Howell post-hoc test indicated that Tier 5 tracts (*M* = $83,834, *SD* = $29,088) are significantly (*p* < 0.001) more affluent than Tier 4 (*M* = $54,283, *SD* = $16,409), Tier 3 (M = $40,932, *SD* = $15,953), Tier 2 (*M* = $42,208, *SD* = $19,464) and Tier 1 (*M* = $49,220, *SD* = $17,737) tracts. However, while there was no significant difference between Tiers 1 and 2, Tiers 1 and 4, or Tiers 2 and 3. there was a statistically significant difference between Tiers 1 and 3 (*p* = 0.018) (Fig 4A).

The second one-way ANOVA was used to determine if there were significant differences in the diversity index between broadband tiers. The Welch’s ANOVA indicated significant group differences, *Welch’s F*(4, 361) = 78.8, *p* < 0.001. Pairwise comparisons of the means using the Games-Howell post-hoc test indicated that Tier 1 tracts (*M* = 9.92, *SD* = 8.63) were significantly less diverse than Tier 2 (*M* = 20.53, *SD* = 21.31), Tier 3 (*M* = 32.08, *SD* = 23.74), Tier 4 (*M* = 30.03, *SD* = 18.16) and Tier 5 (*M* = 31.04, *SD* = 15.69) tracts. However, Tier 3, Tier 4 and Tier 5 tracts were not significantly different from each other. Fig 4B provides a graphical display of the means for each group. A deeper dive into these demographic data (not formally reported) indicated that the profile of Tier 1 tracts is overwhelmingly white, with 60% located in (or immediately adjacent to) Appalachian Ohio. However, there was a contiguous cluster of Tier 1 tracts (*n* = 10) in the East Cleveland, Ohio area that was overwhelmingly black in composition (> 90%). This finding is discussed in the next section.

The third one-way ANOVA was used to determine if there were significant differences in the education levels of the population in each broadband tier. This is important in the context of breastfeeding because mothers with higher levels of formal education often initiate and continue breastfeeding more regularly than mothers with lower levels of formal education [96, 97]. The Welch’s ANOVA indicated significant group differences, *Welch’s F*(4, 350) = 630, *p* < 0.001. Pairwise comparisons of the means using the Games-Howell post-hoc test indicated that Tier 1 (*M* = 13.40, *SD* = 6.83), Tier 2 (*M* = 13.4, *SD* = 6.22) and Tier 3 (*M* = 15.30, *SD* = 9.04) tracts exhibited significantly lower levels of formal education than Tier 4 (*M* = 26.0, *SD* = 13.99) and Tier 5 (*M* = 49.3, *SD* = 17.13) tracts (Fig 4C).

The last suite of one-way ANOVAs was used to determine if Ohio tracts with at least 10/1 Mbps connection speeds and their associated broadband connection density tiers (1, 2, 3, 4, or 5) (Table 4) differ in access to breastfeeding support resources. First, the aggregate shortest path distance required for travel to each breastfeeding support type (IBCLC, LLL or BfUSA meeting, Baby-Friendly Hospital, and WIC) from each tract is used as the measure of access. The Welch’s ANOVA indicated significant group differences, *Welch’s F*(4, 322) = 78.1, *p* < 0.001. Pairwise comparisons of the means using the Games-Howell post-hoc test indicated residents in Tier 5 tracts (*M* = 67.70, *SD* = 56.60) are more proximal to breastfeeding resources than residents in Tier 4 tracts (*M* = 89.60, *SD* = 72.70), Tier 3 tracts (*M* = 120.30, *SD* = 92.30), Tier 2 tracts (*M* = 154.10, *SD* = 106.20) and Tier 1 tracts (*M* = 173.30, *SD* = 117.90) (Fig 4D). In fact, these results hold in a hierarchical fashion for almost all the broadband tiers. This means that Tier 4 tracts are more proximal to breastfeeding resources than Tier 3 tracts, Tier 3 tracts are more proximal than Tier 2 (*p* < 0.001). However, the results indicate that there are no statistically significant differences in proximity between Tier 1 and Tier 2 tracts (*p* = 0.815). Given the spatial distribution of these support resources and Ohio’s 10/1 broadband connection densities, this overall urban bias (Fig 3) might be expected, but it underscores the geographic challenges facing residents in more remote regions when seeking breastfeeding support. Not only are the physical resources more distant, the density of 10/1 broadband connections is highly diminished when compared to their urban peers. An identical result emerged when broadband connection density tiers were compared to the *sum of relative maximum distances* for each tract to access an IBCLC, LLL or BfUSA meeting, Baby-Friendly Hospital, and WIC office (Fig 4E).

The simple takeaway from the ANOVA tests is that tracts classified as Tier 1, 2, and 3 for broadband density, tend to be poorer, less educated and further away from community resources than Tier 4 and 5 tracts. For complete tabular results, see S1 Appendix.

### 4.3 Estimating local access

In an effort to identify tracts that may need some type of intervention to improve both physical and virtual access to breastfeeding support, estimates of local, physical access to support options are required for each Census tract. As detailed previously, using tract centroids as a representative point for estimating access to breastfeeding resources is a viable strategy, but the morphology of tracts in Ohio is quite varied. While relatively small and compact in the major cities (which means the centroids are better representative points), tracts are much larger in the rural portions of Ohio. This means that centroids are poorer approximations of access for the residents in these locales.

**Fig 5:**
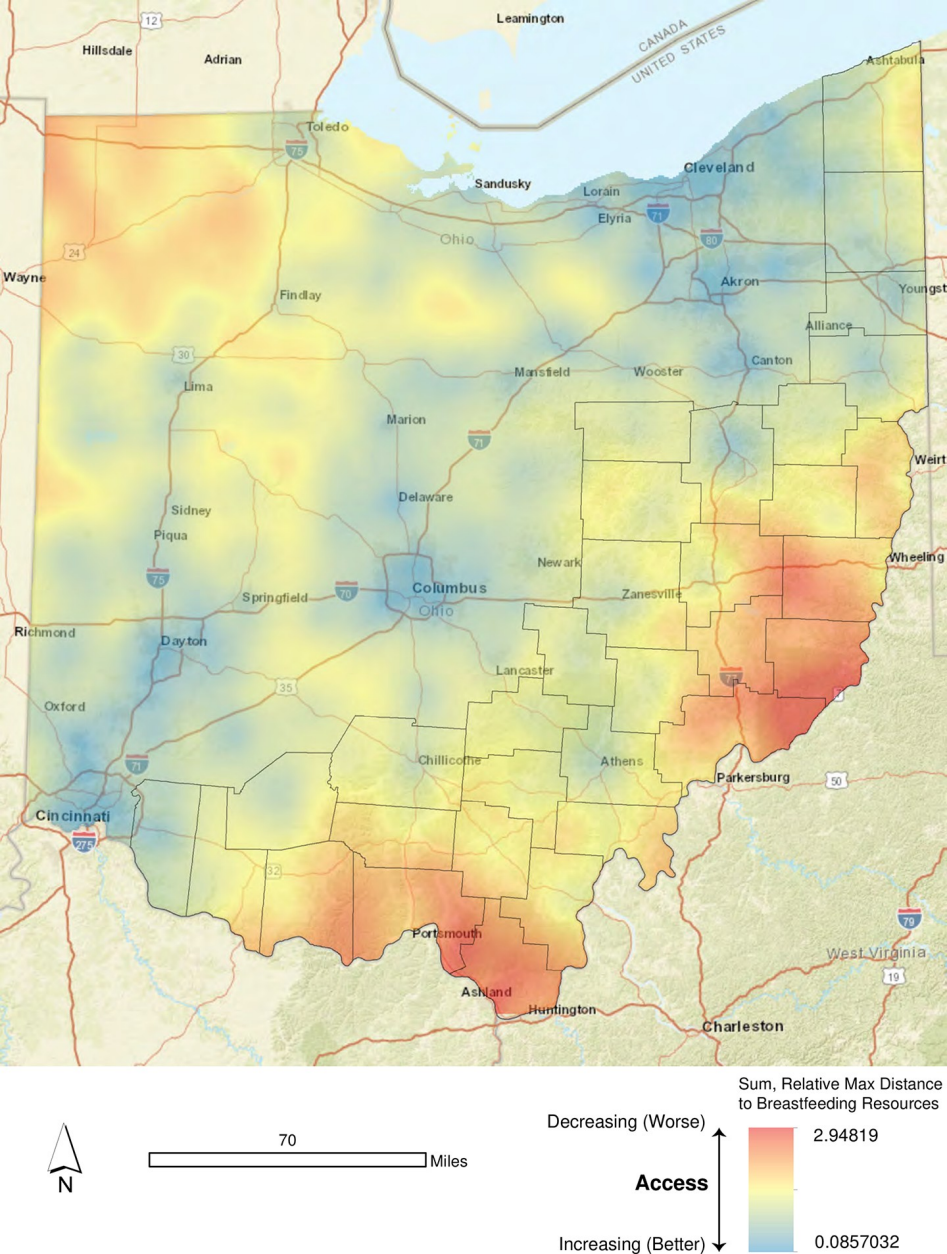
Estimated geographic access to breastfeeding support options.

In an effort to provide a more realistic snapshot of access to breastfeeding resources for all residents in Ohio, we used ordinary kriging to estimate an access surface for the state. Fig 5 displays the results of this effort. We derived the local access surface using the *sum of relative maximum distances* measure detailed previously. Higher (better) levels of access are denoted in cooler shades (e.g., blue) while lower (poorer) levels of access are denoted in warmer shades (e.g., orange). Large portions of Appalachian Ohio and Northwest Ohio exhibited lower levels of access to breastfeeding resources, but this is not strictly a function of interstate highway presence/no presence. Portions of Interstates 71, 75 and 90 traverse several of the most access-challenged regions of the state, where there are significantly fewer breastfeeding support options.

We further refine the results of this analysis through the use of zonal statistics for the generated surface. Specifically, we derived the unique, access values (e.g., *min*, *max*, *mean*) for each surface observation in each tract. Consider, for example, Tract 39059977100, which is located in Guernsey County, Ohio, near Salt Fork State Park. The sum of relative maximum distances for the centroid-based analysis (conducted earlier) is 2.193, but the estimated access surface (derived from ordinary kriging) for that tract ranges from 1.716 to 2.580, with a mean of 2.164. While this mean value is very close to the sum of relative maximum distance for the centroid, the zonal statistics helped to provide a more balanced snapshot of access to breastfeeding resources, especially for larger, morphologically complex tracts.

### 4.4 Identifying potential intervention locations

Next, in an effort to identify locations that may require some type of intervention, the mean access value for each tract is cross-referenced with the broadband connection tiers to highlight locales in Ohio that exhibit compound risk for lack of access to breastfeeding support. Specifically, Stage 1 tracts are those that exhibited mean access values greater than 1 and correspond to Tier 1 or 2 broadband connectivity levels. Stage 2 tracts are those that exhibited mean access values greater than 1.5 and also correspond to Tier 1 or 2 broadband connectivity levels.

The mean access thresholds for identifying Stage 1 and Stage 2 tracts are somewhat arbitrary, but their interpretation is not. Mean access values greater than 1 were found in 525 tracts for Ohio. Stage 1 tracts, which have a mean greater than 1, but less than 1.5, are found in 100 of the 2,915 (3.43%) tracts used for analysis. Stage 1 tracts are home to 79,816 women of child bearing age (20–49). The Stage 1 designation suggests that the effort required to access LLL or BfUSA meetings, an IBCLC, a WIC office, or Baby-Friendly hospital is significantly higher than peer tracts with lower scores. This is amplified in Stage 2 tracts (73 of 2,915; 2.50%), where mean access scores are greater than 1.5. A total of 52,457 women of child bearing age live in these Stage 2 tracts.

From a geographic perspective, many Stage 1 tracts are located closer to the smaller urban centers (e.g., Athens, Chillicothe, Lima, etc.) that are scattered throughout Ohio. This proximity means that while access to breastfeeding resources for Stage 1 tracts is not impossible, these are locations that would benefit from telelactation options for mothers not wanting to make long trips in the car (if personal transportation is available) with an infant/child to reach a support resource. Stage 2 tracts are locations that require significantly *more* travel effort to reach breastfeeding support resources and also exhibit low-levels of broadband connectivity. In these locations, primarily Appalachian Ohio and Northwest Ohio, improved telelactation options are critical. Thus, while both Stage 1 and Stage 2 tracts could be considered strong candidates for some type of policy intervention that either improves physical access to breastfeeding support resources or improves the broadband landscape through provision, pricing, or quality of service, these improvements are absolutely necessary for Stage 2 locations.

## 5. Discussion and conclusion

**Fig 6:**
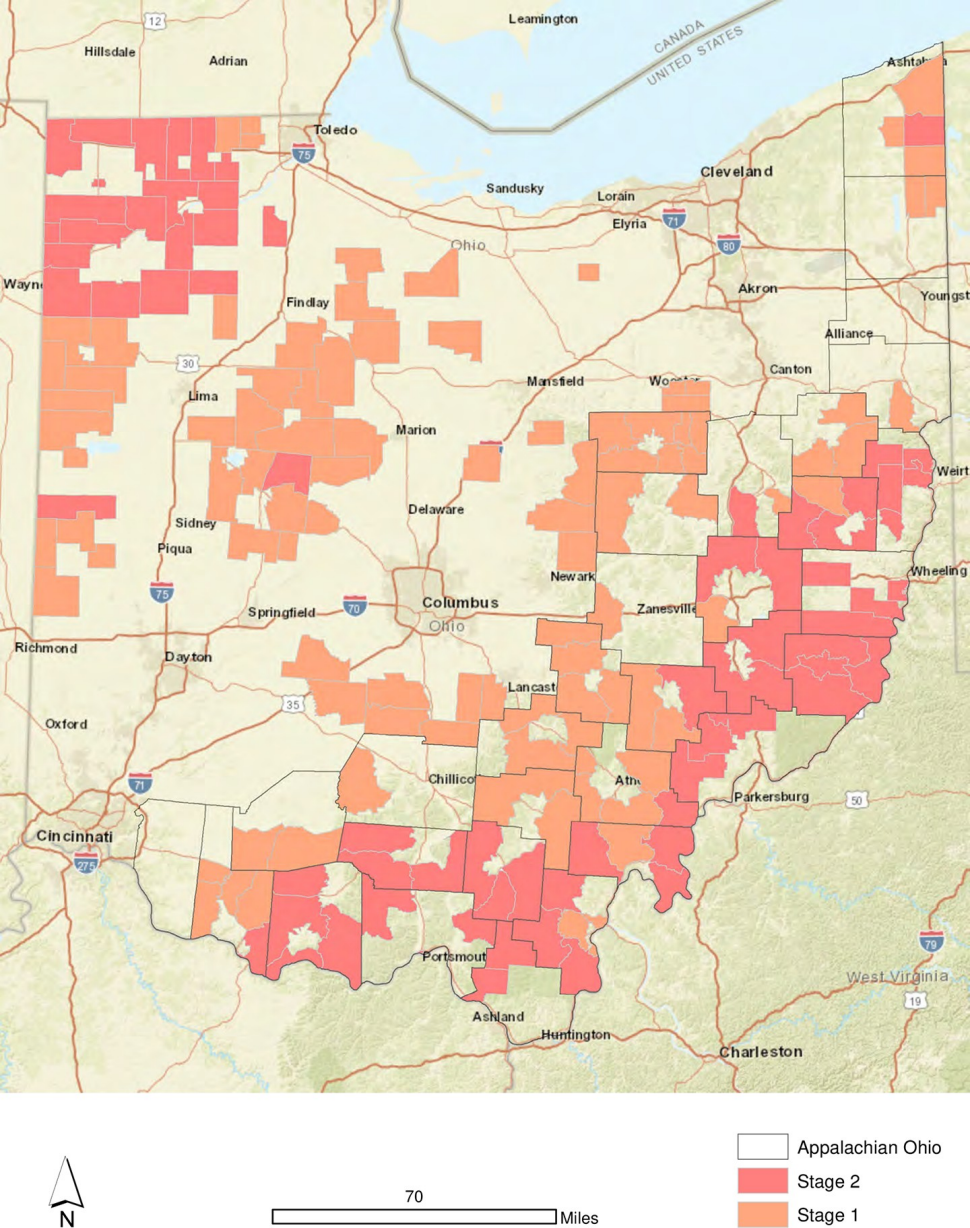
Stage 1 and Stage 2 intervention locations by census tract in Ohio.

There are three facets of the results worth further discussion. First, within the state of Ohio, there is a strong association between the travel distances required to physically access breastfeeding support resources and the density of 10/1 broadband connections. Specifically, the results suggested that locales with the lowest density of broadband connections are also located furthest from community-based breastfeeding support locations such as LLL or BfUSA meetings, Baby-Friendly Hospitals, IBCLCs, and WIC clinics. Further, both the Stage 1 and Stage 2 intervention locations (Fig 6), home to 132,273 women of child-bearing age, tend to be areas of lower socioeconomic status with many found in historically disenfranchised areas such as Appalachian Ohio. All of these factors can lead to lower levels of breastfeeding initiation and duration.

Second, the identification of Stage 1 and 2 tracts and helps to pinpoint locations that are in the most need for intervention. From physical access, to broadband access, or the combination of both, the fusion of data and geocomputational techniques illustrated in this paper provides an ability to flag inequitable distributions of support resources and the communities that need help. While the factors that contribute to a lack of broadband access are widely known, especially in rural areas [68, 71, 72, 98], issues of affordability and socioeconomic status (SES) [99, 100] continue to slow access, availability, and adoption. Further, in many communities, acquiring broadband can be more difficult for residents who cannot speak English (e.g. Hispanic communities), who are not participants in the traditional banking system, and do not have a credit or debit card to pre-register and make the required deposit to start broadband services [70]. These underlying structural inequalities can make breastfeeding support, and success, more difficult to achieve in areas that are already struggling for a host of other reasons.

**Fig 7:**
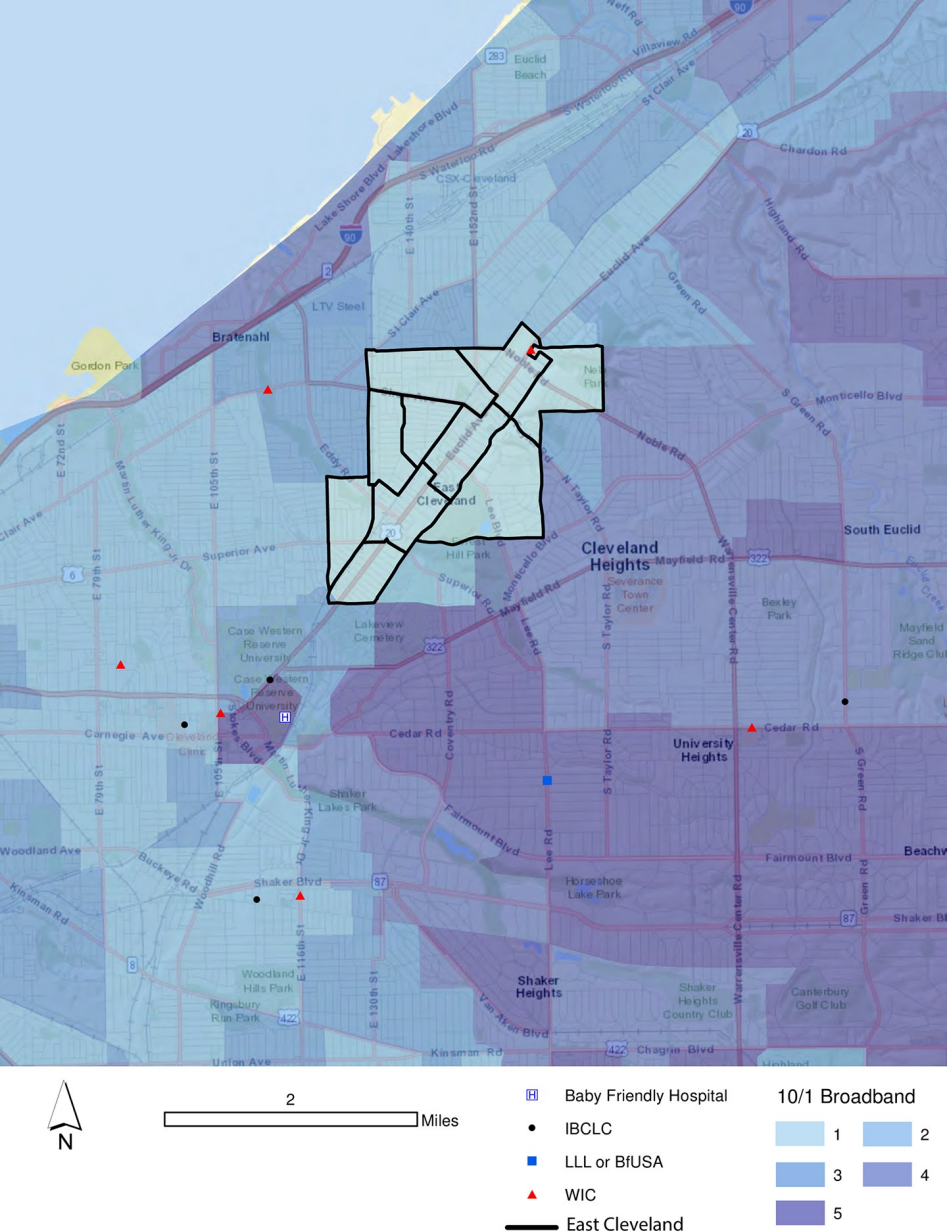
East Cleveland’s physical and virtual access options for breastfeeding support.

While these challenges can seem insurmountable, there are reasons for hope. Consider the case of East Cleveland, Ohio, which is best described as overwhelmingly black in composition (> 90%). East Cleveland was also ranked as the 4^th^ poorest city in the U.S., with a poverty rate of 41.8% in 2018 [101]. Simply put, residents in this community, and many Clevelanders more generally, suffer from digital exclusion [102]. This refers to a combination of deficiencies for residents, including a lack of access to affordable broadband networks and computing hardware, poor literacy for navigating, consuming and producing digital content and no means for troubleshooting when computing devices or broadband connections are not functioning [103]. While this is certainly problematic for residents of East Cleveland, none of these tracts qualified as Stage 1 or Stage 2 intervention candidates in this analysis. Fig 7 highlights the reason why. Not only is there a WIC clinic in East Cleveland, the MacDonald Women’s Hospital, which is Baby-Friendly, is located on Euclid Avenue, less than one kilometer from East Cleveland’s municipal boundary. Further, there is an LLL meeting about 2 km south and several additional WIC clinics and IBCLCs nearby. In short, while there is little doubt that the landscape for telelactation could be improved for mothers in East Cleveland, the existing physical access to local breastfeeding support resources appears sufficient.

However, in many locations throughout Ohio, the situation is reversed. As mentioned previously, some of the smaller urban areas have good broadband, even though the physical access to breastfeeding support resources is relatively poor. The Marietta, Ohio area, which is located on the north bank of the Ohio River in Washington County (Appalachian Ohio) along Interstate 77 is a representative example. There are two WIC clinics (Marietta, OH and Belpre, OH) in the area, but there are no LLL or BfUSA meetings, no Baby-Friendly Hospitals, nor are there any IBCLCs in Marietta. However, the bulk of Census tracts in/around Washington County belonging to Tier 3 and Tier 4 for broadband. This means that telelactation is a viable option for mothers in the region, which is why the tracts for Marietta were not flagged as Stage 1 or Stage 2 intervention candidates. As one moves away from Marietta and into the more rural portions of Appalachian Ohio, breastfeeding support options (both physical and virtual) erode quickly and many nearby tracts are flagged as needing support. In fact, these are the rural/remote locations where telelactation should hold the most promise, but decades of involuntary exclusion and deepening economic and social inequalities lead to a vicious digital cycle [104] where these rural locales are always at least one or two technological generations behind (if not more) for digital connectivity [38].

Third, in an era increasingly defined by social distancing [105], the prospects for telelactation, and telemedicine more generally [106], have never been better. This also includes the delivery of postnatal care, for example, with broadband facilitating the delivery of telecounselling for those experiencing postnatal mental health issues [107]. However, the United States, Australia, the United Kingdom and many other developed nations have significant work ahead of them to make this a reality. In the United States, broadband provision largely remains a private-sector effort. This means that the largest providers (e.g., Spectrum, Verizon, etc.) are looking to rollout infrastructure in areas that will provide the greatest return on investment (ROI). Because teledensities [108] are so much lower in rural areas when compared to urban locales, there is little incentive for providers to allocate broadband infrastructure in such settings. But this need not be the case. There are many “lagging” rural areas that are taking matters into their own hands, including portions of the Texas Hill Country (west of Austin) that historically had very limited broadband options. Cobler [109] details the emergence of a local wireless broadband company that now functions as the largest provider for Johnson City, TX. This is exactly what is needed for Appalachian Ohio and the good news is that progress is being made. The Appalachian Regional Commission recently announced a $2.5 million grant for the Buckeye Rural Electric Cooperative in Gallipolis, Ohio to install 168 miles of fiber to create a backbone network for underserved areas in Athens, Gallia, Jackson, Lawrence, Meigs and Vinton counties [110], many of which are home to Stage 1 and 2 intervention locations.

It is equally important to facilitate better physical access to breastfeeding support resources in rural areas. While the presence of WIC clinics certainly help, alternative forms of support such as LLL and BfUSA remain important for mothers seeking peer-support. Neither organization has direct control over where and when meetings form because peer-support is fueled by volunteers. This is not to say that rural and remote areas are devoid of LLL or BfUSA meetings, but similar to the case for Ohio, the bulk of their service footprint exists in the urban areas of the United States [111].

### 5.1 Limitations

There are several limitations and dynamics to the analysis and results worth noting. First, the access landscape detailed for Ohio represents a relatively conservative snapshot. In part, this is attributed to the presence of edge effects. Specifically, Ohio is not an “island”, where any/all breastfeeding support options stop at the border. For example, it is possible for mothers located in Northwest Ohio to obtain support from neighboring states and/or cities, including places like Fort Wayne, Indiana. However, as mentioned previously, this likely requires some form of personal transportation and long travel times–often during inclement weather. It also takes mothers away from their local social network(s), which are an important part of the community support matrix [30]. Further, while LLL or BfUSA meetings are free and all are welcome to attend, giving birth in a Baby-Friendly hospital, out of state, may not be possible–especially if the hospital is not within one’s insurance network. Costs would certainly escalate.

Second, the breastfeeding support landscape is constantly changing, as are the technologies and associated platforms that one might use for telelactation. As a result, the results of this analysis should not be considered binding for the state of Ohio. There is certainly room for change and/or improvement, especially when considering intervention policies, education, direct investment, public/private partnerships or other strategies for closing the breastfeeding support divide.

Finally, the Stage 1 and 2 tracts flagged for interventions would certainly require more analysis before any actions were taken. In many locales, there may be support resources that were missed in this analysis. For example, portions of Appalachian Ohio are home to high density Amish settlements [112]. In fact, all of the tracts in Holmes County were flagged as Stage 1 intervention candidates, but the Amish populations in Holmes County have no interest in broadband connections, telelactation, Baby-Friendly Hospitals or IBCLCs. Amish populations tend to favor home-births [113], breastfeeding is the norm [114], and breastfeeding wisdom is passed on from kin or the larger community.

In short, while the methodological framework detailed in this paper helps identify communities needing intervention, especially those where population densities are lower (i.e., rural and remote areas), nuances and local context exist. Some of the areas flagged as Stage 1 and 2 intervention areas may not have significant problems (e.g., Amish communities), while others that were not flagged (e.g., East Cleveland) still face challenges. In a world increasingly defined by social distancing, these dynamics are important to consider when developing intervention strategies.

### 5.2 Conclusion

Telelactation is a promising evolution in breastfeeding support. Not only can mothers obtain support from the comfort of their own homes, mothers located in rural or remote locations where lactation professionals may not be available can receive high-quality support when they need it. In addition, telelactation can cost less than face-to-face appointments with an IBCLC and it helps mothers develop their skills independently, improving self-efficacy [54]. The results of this paper suggest that both physical access and virtual access to breastfeeding support resources remain a challenge, especially for rural and remote areas of Ohio. Specifically, while mother’s in Ohio’s urban areas often benefit from a wide range of support resources, including LLL and BfUSA meetings, IBCLCs, Baby-Friendly Hospitals and WIC clinics, as well as high-quality broadband connections, this is not true for most of Northwest and Appalachian Ohio. Many of the Census tracts located in these regions are not proximal to physical resources and local broadband connectivity is sparse, at best. These inequities are not insurmountable, but it will take a concerted effort to mitigate them. This includes investments in broadband networks to facilitate telelactation, especially in areas where breastfeeding support resources and allied healthcare infrastructure is thin. Finally, it is important to acknowledge the generalizability of the approach detailed in this paper. The data requirements are relatively modest and the associated methodological framework is easily repeatable. The ability to pinpoint communities needing intervention can help guide investment decisions for improving broadband and/or access to physical support resources and may provide a pathway for positively impacting the delivery of breastfeeding support in Ohio and beyond.

## Supporting information

S1 Appendix (TIF)
