# Supplementary figures and images for: The complex geographies of telelactation and access to community breastfeeding support in the state of Ohio
Source: PLoS One. 2020 Nov 24;15(11):e0242457. doi: 10.1371/journal.pone.0242457 (PMC7685454; doi:10.1371/journal.pone.0242457)

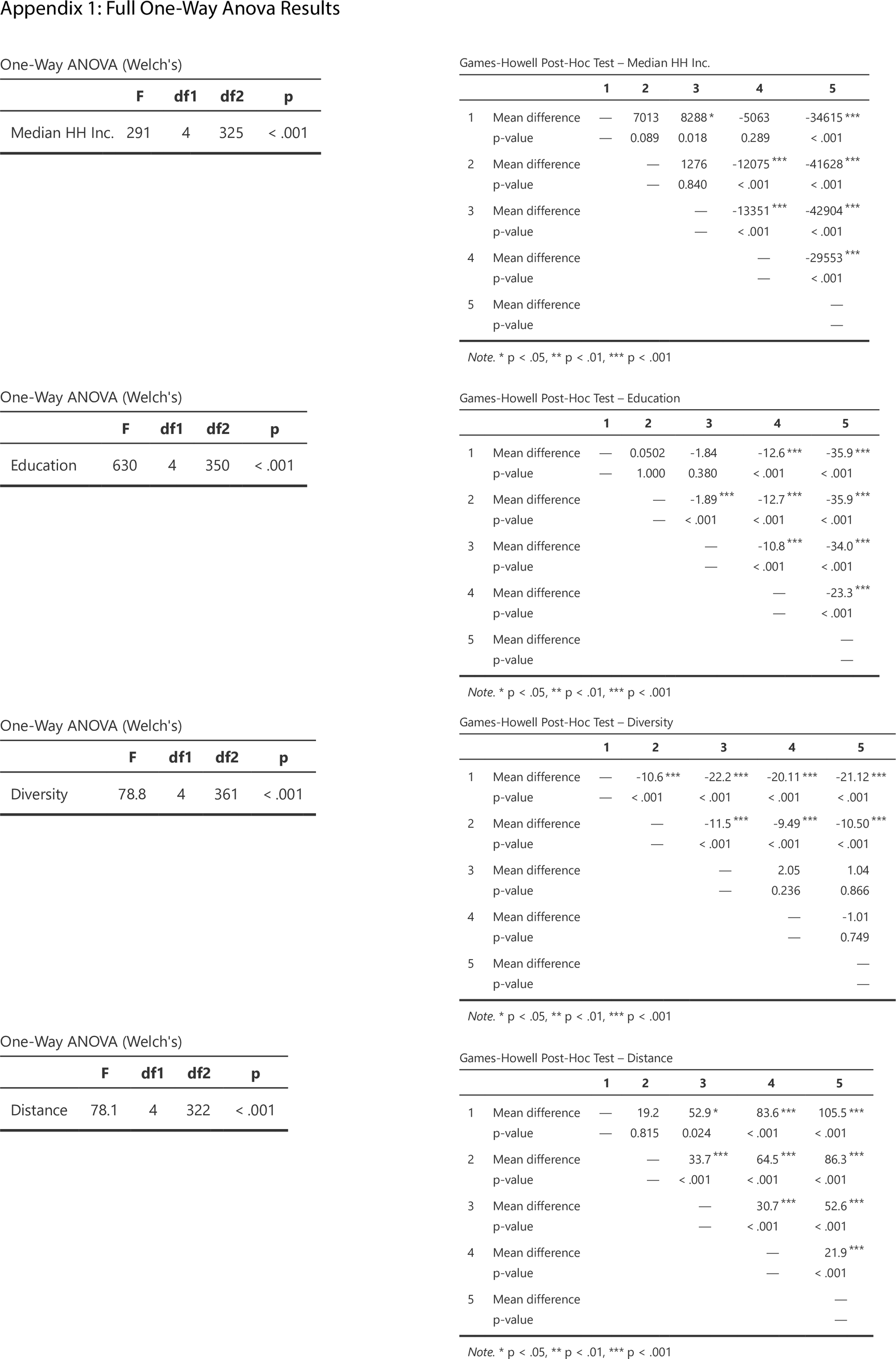

Supplement: S1 Appendix — (TIF) [file pone.0242457.s001.tif]
